# Mesenteric Adipose Tissue as a Modulator of Intestinal Inflammation: Mechanistic Insights and Model Systems in Crohn's Disease

**DOI:** 10.1002/eji.70222

**Published:** 2026-06-17

**Authors:** Toka Omar, Carl Weidinger, Britta Siegmund

**Affiliations:** ^1^ Department of Gastroenterology, Infectious Diseases and Rheumatology Charité ‐ Universitätsmedizin Berlin, Corporate Member of Freie Universität Berlin and Humboldt‐Universität zu Berlin Berlin Germany; ^2^ Cluster of Excellence Immunoprecept Charité ‐ Universitätsmedizin Berlin Berlin Germany

## Abstract

Inflammatory bowel disease, including Crohn's disease (CD) and ulcerative colitis, is becoming increasingly prevalent. Obesity, specifically visceral adiposity, has been identified as a significant risk factor for disease recurrence and complications. A hallmark of CD is the development of “creeping fat”, which is characterized by adipocyte hyperplasia and increased adipokine secretion. Under homeostatic conditions, adipose tissue plays a key role in immune regulation through the secretion of adipokines, such as leptin and adiponectin, which modulate JAK/STAT, AMPK, and NF‐kB pathways in immune and epithelial cells. However, in obesity and under metabolic stress, mesenteric adipose tissue undergoes immune remodelling marked by macrophage polarization, Th1/Th17 differentiation, and fibroblast reprogramming driven by TNFα, IL‐6, and TGF‐β1. These changes promote sustained cytokine production, extracellular matrix deposition, and fibrostenotic remodelling of the intestinal wall. This review highlights current evidence linking mesenteric adipose tissue and creeping fat immunometabolism to intestinal inflammation in CD. We outline key cellular and signalling mechanisms that connect mesenteric adipose to mucosal immune dysregulation and discuss emerging experimental models.

## Graphical abstract

This review explores how mesenteric adipose tissue, particularly creeping fat, contributes to intestinal inflammation through dysregulated adipokine signalling. We explore how metabolic stress alters immune‐adipose interactions and promotes disease progression. Insights from mechanistic studies highlight mesenteric adipose tissue as a key driver of inflammation and a potential therapeutic target.

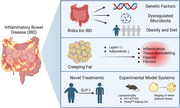

**Abbreviations**
CD: Crohn's disease
CF: creeping fat
*db/db* mice: leptin receptor knockout mice
DSS: dextran sodium sulfate
GLP‐1: glucagon‐like peptide 1
HFD: high‐fat diet
IBD: inflammatory bowel disease
LepR: leptin receptor Ob‐Rb
MetS: metabolic syndrome
*ob/ob* mice: leptin‐deficient mice
Reg: recombination activation gene
Scid: severe combined immunodeficient mice
TNBS: 2,4,6‐trinitrobenzene sulphonic acid
Treg: regulatory T cells
VAT: visceral fat

## 1 Introduction

Inflammatory bowel disease (IBD) is a chronic, immune‐mediated disorder characterized by inflammation of the gastrointestinal tract and encompasses both ulcerative colitis and Crohn's disease (CD) [1]. CD is characterized by transmural inflammation that can affect any region of the gastrointestinal tract in a discontinuous pattern. Inflammation in ulcerative colitis is commonly restricted to the colon and rectum in a continuous pattern and is limited to the mucosa and submucosa [1]. IBD is a debilitating chronic disease with uncertain aetiology; the development of IBD is more likely to occur in individuals with a primarily susceptible genetic background, often in association with dysregulated immunity, microbial dysbiosis, and environmental triggers [2].

### 1.1 IBD and Its Comorbidities

Obesity is considered a low‐grade inflammatory state that has been identified as a risk factor for numerous autoinflammatory conditions, including rheumatoid arthritis, systemic lupus erythematosus, and IBD [3, 4, 5, 6, 7]. The recent rapid increase in obesity worldwide has prompted several studies to examine the relationship between obesity and IBD disease activity and outcome. Despite their limitations in scope, these studies offer valuable insights into the interplay between obesity and IBD disease progression.

Preclinical models demonstrate that obesity exacerbates colitis following dextran sodium sulfate (DSS) or 2,4,6‐trinitrobenzene sulphonic acid (TNBS) treatment by inducing oxidative stress in the colon, elevated cytokine production (TNFα and IL‐6), and dysregulated mucosal immune responses [8, 9]. Clinically, obesity, particularly increased levels of visceral fat (VAT), correlates with a more complex CD phenotype characterized by stricturing and/or fistulizing disease, increased risk of postoperative CD recurrence, increased risk of developing postoperative complications such as wound healing (OR 1.7), and reduced use of minimally invasive colorectal surgery due to increased technical difficulty [10, 11, 12]. Mechanistically, VAT in obese people is enriched in pro‐inflammatory immune cells, such as M1 classically activated macrophages that secrete in large amounts TNFα, IL‐6, IL‐12, IL‐1β, as well as nitric oxide [13].

### 1.2 Adipose Tissue and Immunometabolism

These clinical associations highlight the need to understand how adipose tissue mechanistically contributes to intestinal inflammation. Adipose tissue depots are heterogeneous in their immune landscape and metabolic activity, with specialized functions in tissue homeostasis and inflammation. Subcutaneous adipose tissue is located under the skin and is typically associated with lipid storage and exhibits a comparatively anti‐inflammatory profile. In contrast, visceral adipose depots surround internal organs and display a higher metabolic activity with a heightened tendency for inflammatory cytokine production [14]. Within visceral fat, mesenteric adipose tissue represents a unique compartment due to anatomical and vascular association with the intestine. Therefore, we here specifically focus on the role that mesenteric adipose tissue plays in immune homeostasis.

White adipocytes make up to 50% of the cell composition, which is responsible for regulating and maintaining lipid storage as well as producing several hormones and cytokines, including leptin and adiponectin [15]. In addition, several different types of immune cells infiltrate the abdominal adipose tissue, including neutrophils, macrophages, T cells, and NK cells [16]. However, metabolically healthy adipose tissue tends to have minimal immune infiltration, mainly controlled through IL‐10 signalling by regulatory T cells (Tregs) [17].

**FIGURE 1:**
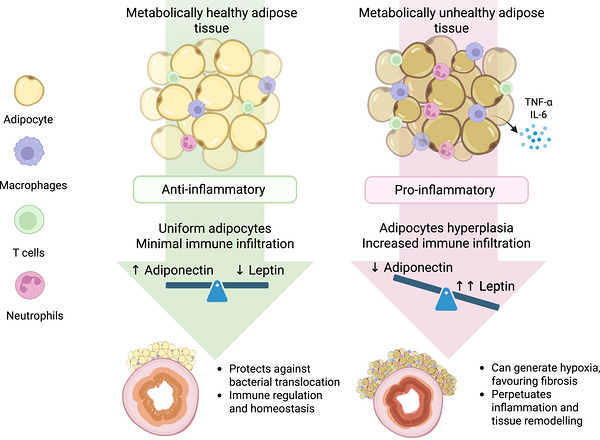
Differences between lean and obese mesenteric adipose tissue on intestinal inflammation. In a lean individual, adipose tissue is in an anti‐inflammatory state due to the low secretion levels of leptin and high levels of adiponectin. Mesenteric fat protects against bacterial translocation while also regulating immune function to maintain homeostasis [28]. However, in an obese individual, adipocytes undergo hyperplasia and hypertrophy, leading to adipokine dysfunction with higher secretion levels of leptin. There is also higher immune infiltration, specifically macrophages, which secrete IL‐6 and TNFα. This not only exacerbates intestinal inflammation but can also lead to fibrosis by generating hypoxia and tissue remodelling [29].

In the case of obesity, mesenteric adipose tissue undergoes significant adaptive remodelling characterised by increases in adipocyte size (hypertrophy), number (hyperplasia), and shifts in immune cellular composition (Figure 1). This expansion is frequently not matched by sufficient vascularisation, resulting in localised hypoxia within the tissue. Hypoxic conditions, together with nutrient stress, promote a dysfunctional adipocyte phenotype in which hypertrophic adipocytes secrete elevated levels of pro‐inflammatory adipokines, including leptin and resistin, while the production of the anti‐inflammatory adiponectin is reduced [18]. This altered adipokine profile promotes activation of NF‐κB and JAK/STAT signalling in tissue resident macrophages and T cells, sustaining systemic cytokine production (TNFα, IL‐1β, IL‐6) that can prime the intestinal mucosa for heightened inflammatory responses [19, 20]. Adipose tissue dysfunction in obesity also leads to recruitment of M1‐polarized macrophages through CD8^+^ effector T cells, resulting in local secretion of IFNγ and IL‐17, cytokines known to drive epithelial injury and mucosal barrier disruption [21].

### 1.3 Role of Mesenteric Adipose Tissue in IBD

These obesity‐associated changes in adipose tissue provide a mechanistic basis for its contribution to intestinal inflammation in IBD. In CD, mesenteric adipose tissue undergoes extensive remodelling into so‐called “creeping fat” (CF), which was first described in 1932 [22]. CF is characterised by the hyperplasia and hypertrophy of the mesenteric fat adjacent to the inflamed section of the small intestine, fibrosis, bacterial translocation, and accumulation of activated immune cells [16, 23]. These features distinguish creeping fat not only from subcutaneous adipose depots but also from other visceral fat, which differ in developmental origin and immune landscape. Recognising this heterogeneity is crucial for interoperating experimental obese models and understanding the specific contribution of mesenteric adipose tissue to intestinal inflammation in CD.

**FIGURE 2:**
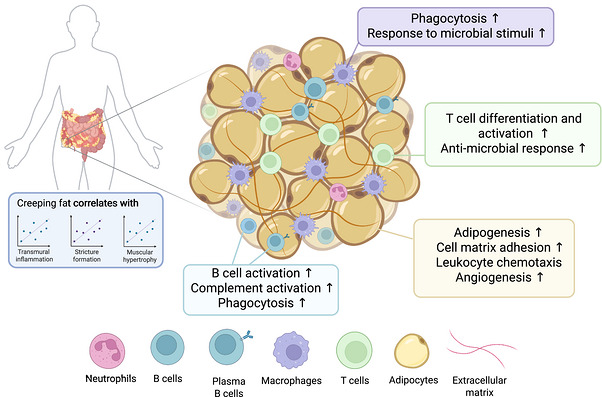
Creeping fat in Crohn's disease (CD). Creeping fat (CF) refers to the expansion of mesenteric fat around inflamed and fibrotic intestinal tissue. CF correlates with transmural inflammation, muscular hypertrophy, and stricture formation. Several studies identified the cell composition of CF in hopes of discovering new therapeutic targets for CD. Some of the important cell clusters in CF include mesenchymal stem cells with high adipogenic capacity, endothelial cells, innate immune cells such as neutrophils, classical and nonclassical macrophages, and DCs, and adaptive immune cells such as B cells, T cells, and IgG^+^ plasma cells. Macrophages specifically were found to play an important role in promoting adipogenesis and the formation of creeping fat. GO analysis of upregulated genes in CF compared with healthy mesenteric fat highlights signatures of fibrosis, angiogenesis, and innate immune response to bacterial stimuli.

At a molecular level, CF exhibits increased levels of TNFα, IL‐6, IL‐1β, and MCP‐1, accompanied by increased levels of leptin and reduced adiponectin secretion, thus shifting adipose tissue toward a pro‐inflammatory state, as shown in Figure 2. These adipokines and cytokines activate NF‐kB, STAT3, and MAPK pathways in infiltrating macrophages and T cells, sustaining macrophage polarization and Th1/Th17 cell differentiation. Consequently, CF correlates with transmural inflammation, muscular hypertrophy, and stricture formation [24].

Despite these mechanistic insights, the functional role of CF in disease progression remains debated. Surgical evidence is inconsistent: some studies report that extended mesenteric excision and concomittant partial removal of CF is associated with reduced postoperative recurrence rates [25], whereas findings from the prospective SPICY trial and other investigations have not demonstrated a significant benefit from partial CF resection [26, 27]. These discrepancies underscore the need for further mechanistic studies to clarify the role of CF in the pathogenesis and progression of CD.

Cellular and transcriptomic analyses have highlighted the immunofibrotic architecture of CF. Single‐cell and bulk RNA sequencing studies have identified a predominance of pro‐inflammatory and pro‐fibrotic preadipocyte clusters that highly express *MIF*, *IL6*, *TNFAIP3*, and *CCL2*, as well as extracellular matrix genes such as *COL1A1, FN1*, and *MMP9* [23]. Multiple immune clusters enriched in antigen‐presenting macrophages (CD68^+^, HLA‐DR^−^), B cells, and IgG^+^ plasma cells show activation signatures in response to microbial exposure. Ligand–receptor analysis revealed dense cytokine networks involving CCL2/CCR2, LIGHT/LTβR, PDGF/PDGFR, MIF/CD74, and SEMA3/NRP1 axes, which collectively promote leukocyte infiltration, fibroblast proliferation, and extracellular‐matrix deposition [30].

Recent mechanistic evidence further implicates that CF‐derived fibroblasts act as active drivers of intestinal fibrosis. Bauer‐Rowe et al. identified a population of mechanosensitive fibroblasts within CF that respond to adipose expansion and matrix stiffening via YAP/TAZ‐dependent signalling. These fibroblasts exhibit enrichment of *ACTA2, COL1A1*, and *FN1*, and secrete TGFβ1 and CTGF, reinforcing myofibroblast differentiation in the adjacent muscularis propria. Co‐culture and organoid models demonstrated that mechanical cues from expanding mesenteric fat activate fibroblast‐macrophage crosstalk, enhancing IL‐6 and MCP‐1 production and promoting collagen deposition [31].

In addition to mechanical cues, emerging evidence implicates the gut microbiome as a driver of CF formation. Viable bacteria such as *Escherichia coli* and Bacteroides/Prevotella spp. [28] and *Clostridium innocuum* were found in the adipose tissue of DSS‐treated mice and CD patients, respectively [23]. Microbial translocation and recognition by adipose resident macrophages via TLR2/TLR4 and NOD2 pathways stimulate IL‐1β and TNFα, reinforcing local immune cell activation and adipose tissue remodelling. This microbial‐adipocyte feedback loop may contribute to sustained adipocyte inflammation and CF maintenance.

Collectively, these findings position CF as an immunometabolic hub integrating microbial sensing, adipokine‐immune signalling, and fibroblast mechanotransduction.

#### 1.3.1 Leptin

Among the adipokines dysregulated in CF, leptin has emerged as a key mediator of immune activation and has been shown to play a pivotal role in regulating metabolism and immunity in a context‐dependent manner. It was initially characterized as a satiety hormone due to its inhibitory effect on appetite [32]. Lord and colleagues provided the first evidence that leptin is key for T cell proliferation and can thus reverse starvation‐induced immunosuppression [33]. This role of leptin is facilitated by the expression of the long isoform of the leptin receptor Ob‐Rb (LepR) on the surface of immune cells [34, 35]. LepR activation can induce STAT3, ERK1/2, and PI3K signalling in a JAK2‐dependent manner [36]. Leptin directly augments the expression of activation markers (CD69, CD25, CD38, and CD71) in human monocytes in vitro [37], and it has been observed to exhibit a dose‐dependent stimulation of pro‐inflammatory cytokine production on murine monocytes [38].

This is also highlighted in the leptin‐deficient model of *ob/ob* mice. These mice exhibit reduced numbers of CD8^+^ intraepithelial T cells, altered intestinal inflammation, and lower levels of inflammatory mediators in models of acute and chronic DSS‐, oxazolone‐, and TNBS‐ induced colitis [39, 40]. These effects could be reversed by leptin replacement [39]. Furthermore, in a T cell transfer model of colitis, naïve LepR‐deficient CD4^+^ T cells resulted in a delayed and attenuated intestinal inflammation when compared with mice receiving WT CD4^+^ T cells [41], highlighting leptin's importance in the development and function of pro‐inflammatory T cells. LepR‐deficient CD4^+^ T cells display impaired development of IL‐17‐producing Th17 cells both in vitro and in vivo [42].

To further dissect the role of adipose‐derived signals, lipodystrophy provides a model for investigating the absence of adipose‐derived immune and metabolic cues. Recently, we demonstrated that mice that lacked adipose tissue featured an attenuated immune compartment, including a significant reduction in splenic NK cells and intestinal CD8^+^ T cells, alongside an improved epithelial barrier integrity at steady state. In addition, the absence of adipose tissue protected lipodystrophic mice against chronic DSS‐induced colitis, evidenced by reduced histopathological inflammation, decreased pro‐inflammatory cytokine production, and enhanced epithelial barrier integrity. This phenotype was associated with decreased pro‐inflammatory T cell phenotype in both systemic and intestinal compartments, indicating that adipose‐derived signals are required to sustain full inflammatory responses in the gut. To specifically address the role of leptin, allogeneic transplantation of adipose tissue was performed. Transplantation of leptin sufficient wild‐type fat but not leptin‐deficient *ob/ob* fat led to a partial rescue of basal leptin levels and exacerbated intestinal inflammation with an increase in Th1 and Th17 immune responses in the colon, consistent with leptin‐driven STAT3‐dependent T cell activation [43].

The role of leptin in T cell homeostasis is also evidenced clinically by the heightened susceptibility to infections in individuals with congenital leptin deficiency or with generalized loss of adipose depots due to lipodystrophy. This can mostly be attributed to a lack of T cell proliferation and, consequently, an insufficient immune response [33, 44]. Leptin promotes the proliferation of naive and memory T cells by increasing Th1 and suppressing Th2 cytokine production [33], while limiting the expansion of Tregs, as demonstrated by increased Treg proliferation in leptin‐deficient mice [45]. These immunomodulatory effects of leptin may help explain its role in exacerbating auto‐inflammatory conditions.

Despite these findings, the role of leptin in intestinal inflammation remains context dependent, with some reporting pro‐inflammatory effects and others suggesting protective functions (Table 1). These discrepancies underscore the need for further mechanistic studies to clarify leptin's context‐dependent functions within the intestinal immune environment.

**TABLE 1:** Mouse models to study the function of leptin in intestinal inflammation.

Mouse model	Leptin protective or pro‐inflammatory?	Model of inflammation	Results
Leptin‐deficient *ob/ob*	Protective	Acute pancreatitis	Supplementation with leptin alleviated intestinal injury in obese *ob/ob* mice by improving intestinal permeability [45]
	Pro‐inflammatory	T cell transfer colitis	*Scid* mice were more resistant to transfer colitis induced by naïve T cells from leptin receptor‐deficient (Ob‐Rb) (*db/db*) mice [41]
		T cell transfer colitis	Naïve CD4^+^ T cells from Cd4 (Δlepr) animals were unable to trigger colitis when adoptively transferred into Rag1^−/−^ host mice [42]
		Acute/chronic DSS, TNBS, and oxazolone‐induced colitis	Reduced severity of intestinal inflammation and less pro‐inflammatory cytokines in acute/chronic DSS, TNBS, and oxazolone colitis, which could be reversed with recombinant leptin injections [39, 40]
Exogenous leptin treatment in wild‐type mice	Protective	Acute DSS‐induced colitis	Protected from DSS‐induced colitis through reinforced intestinal barrier integrity with increased expression of tight junctions [52]
	Pro‐inflammatory	Acute/chronic DSS, TNBS, and oxazolone‐induced colitis	Leptin‐treated WT mice had worsened colitis, with more weight loss, higher inflammation histological scores, and shorter colons [39]
High‐fat diet (HFD)	Protective	Acute DSS‐induced colitis	HFD‐fed mice had higher levels of leptin and were protected from DSS‐induced colitis by reinforcing the colonic barrier function [53]
	Pro‐inflammatory	Acute TNBS‐induced colitis	HFD mice had more severe colitis following DSS or TNBS treatment by inducing oxidative stress in the colon [8]

Abbreviations: *ob/ob*: leptin‐deficient mice; DSS: dextran sodium sulfate; TNBS: 2,4,6‐trinitrobenzene sulphonic acid; *Scid*: severe combined immunodeficient mice; *db/db*: leptin receptor knockout mice; lepr: leptin receptor (ObRb); Rag: recombination activation gene, HFD: high‐fat diet.

#### 1.3.2 Adiponectin

Adiponectin is another adipokine secreted by white adipose tissue and is responsible for promoting insulin sensitivity in the liver while stimulating fatty acid oxidation in skeletal muscle [46]. Unlike leptin, adiponectin is known for its anti‐inflammatory properties as it downregulates inflammatory mediators such as TNFα and IL‐6 while promoting anti‐inflammatory markers such as IL‐10 and arginase1 in macrophages [47, 48]. It has also been shown to suppress immune cells such as neutrophils and gamma‐delta T cells [49, 50]. A recent paper showed that adiponectin was able to reduce immune checkpoint inhibitor‐induced inflammation without inhibiting antitumour immunity in mice treated with DSS and immune checkpoint inhibitors [51].

Two studies provided evidence that adiponectin knockout mice exhibited more severe colitis, increased immune cell infiltration, and an exaggerated pro‐inflammatory cytokine profile following DSS treatment [54, 55]. Conversely, another study showed that adiponectin deficiency was associated with protection from DSS‐ or TNBS‐induced colitis [56]. Based on those studies, the role of adiponectin remains unclear and requires further examination.

### 1.4 Obesity Management in IBD

Given the critical role that obesity plays in intestinal inflammation, there is an urgent need to address the rise of obesity in IBD patients. A plethora of approaches to weight reduction and the reversal of insulin resistance in overweight and obese individuals have been developed. Recently, glucagon‐like peptide 1 (GLP‐1) has gained significant scientific attention for its potential dual role in weight loss and immune modulation, as shown by its ability to significantly decrease inflammation through the reduction of CRP and TNFα [57]. It has been demonstrated to mediate direct effects on many cells, due to the expression of the GLP‐1 receptor (GLP‐1R) on the surface of many immune cells [58]. Yusta et al. [59] showed that GLP‐1R expression was enriched in intestinal epithelial lymphocytes and that Glp1r^−/−^ mice exhibited increased severity of DSS‐induced colitis. Additionally, the administration of GLP‐1 to WT mice was shown to increase the expression of immunomodulatory and antimicrobial genes, suggesting that GLP‐1 treatment may assist in regulating intestinal inflammation [59]. Beyond its metabolic effects, these findings suggest that GLP‐1 signalling may modulate adipose‐immune crosstalk and intestinal inflammation.

Initial studies suggest that patients with IBD receiving antidiabetic GLP‐1‐based therapies exhibit a reduced risk of adverse IBD‐related events. These include a lower likelihood of requiring oral corticosteroids, initiation of anti‐TNF therapy, or hospitalization [60, 61], indicating a potential protective effect of GLP‐1 signalling in the context of IBD. A recent retrospective study demonstrated that there was no significant difference in the risk of steroid use or any‐cause hospitalization between the semaglutide‐treated IBD group and the control group, suggesting that semaglutide is effective as a weight loss treatment in patients with IBD and obesity [62]. These findings underscore the significance of incorporating weight reduction strategies as a component of IBD management; however, more research is needed on the long‐term effects of such weight loss aids on IBD disease progression.

### 1.5 New Models to Study the Role of Adipose Tissue

To elucidate how obesity and adipocyte dysfunction contribute to CD pathogenesis, refined experimental models are required to capture the cellular and molecular complexity of fat–gut crosstalk.

#### 1.5.1 Adipose Tissue Browning as an Immunometabolic Model

Beyond energy regulation, adipose browning provides a potential modulator of immune regulation [63]. Recent research suggests that the browning of white adipose tissue could protect against obesity and its adverse consequences [64]. In CD, mesenteric adipose tissue depots showed a conversion of white to beige adipose tissue together, accompanied by high *UCP1* expression, correlating with systemic elevation in microbiota‐derived succinate [65]. Succinate signals through SUCNR1 receptors on macrophages and adipocytes, promoting the production of pro‐inflammatory cytokines such as TNFα and IL‐6 via HIFα‐dependent pathways. This observation indicates that microbiota‐derived metabolites can promote adipose tissue remodelling, which in turn may amplify intestinal immune activation. Investigating the browning of adipose tissue within this framework could provide critical insights into how metabolic reprogramming of adipose tissue contributes to the progression of CD [65]. This is increasingly important as recent studies suggest that weight loss medications such as GLP‐1 can regulate fat browning [66, 67], thus positioning adipose browning as a potential link between microbial metabolites, adipose remodelling, and intestinal immune activation in CD and a potential therapeutic target in CD.

#### 1.5.2 Lipodystrophy and Loss‐of‐Function Models

Lipodystrophy includes rare disorders characterized by complete or partial loss of adipose depots, thus acting as a loss‐of‐function model. Similar to obesity, lipodystrophic patients suffer from metabolic complications, including insulin resistance, hypertriglyceridemia, and dyslipidaemia [68]. While clinical data regarding lipodystrophy are still in their nascent stages, our group demonstrated that leptin administration induced inflammation in a TNFα‐dependent manner in a patient with combined acquired generalized lipodystrophy and CD. Specifically, leptin treatment led to an increased TNFα production in CD4^+^, CD8^+^ T cells, and natural killer cells, as well as an enhanced perforin expression in CD8^+^ T cells, thus demonstrating how adipokine deficiency and replacement reshape mucosal immune responses [69].

Furthermore, an inducible ablation of adipocytes in FAT‐ATTAC (fat apoptosis through targeted activation of caspase 8) mice enables a mechanistic dissection of adipose tissue contribution to intestinal pathology [70]. In a collaborative study, Liu et al. [71] demonstrated that depletion of abdominal fat in DSS‐treated mice led to a reduction in muscularis propria thickness without affecting mucosal inflammation. These findings suggest that signals derived from adipose tissue contribute to stricture and fibrosis formation independently of classic cytokine‐driven inflammation, providing a potential mechanistic explanation for the association between high abdominal fat and increased stricture formation in patients with CD.

## 2 Conclusion

Mesenteric adipose tissue and specifically creeping fat actively contribute to intestinal inflammation and metabolic regulation, and its dysfunction can exacerbate both inflammatory and fibrotic processes. Elucidating the mechanisms by which mesenteric adipose tissue drives intestinal inflammation is therefore critical for identifying novel therapeutic targets and for mitigating the impact of obesity on IBD.

## Author Contributions

T.O. performed the literature analysis and wrote the manuscript. C.W. and B.S. provided guidance, contributed to interpretation, and critically revised the manuscript. All authors approved the final version.

## Funding

This work was funded by the German Research Foundation We 5303/3‐2 to CW, SFB‐TRR 241 (project‐ID 375876048) B01 to BS and CW, A09 to CW, SFB‐TRR 412 (Grant ID: 535081457) A05 to BS and CW, CRU 5023 (project‐ID: 50474582), CRC 1449‐B04 (project‐ID: 431232613); CRC 1340‐B06 (project‐ID 372486779), SI749/14‐1 (project‐ID: 418055832) all to BS.

## Conflicts of Interest

Britta Siegmund served as a consultant for AbbVie, AltruBio, Abivax, Boehringer Ingelheim, Bristol Myers Squibb, Dr. Falk Pharma, Endpoint Health, Eli Lilly, Galapagos, Janssen, Johnson&Johnson, Materia Prima, MSD, Pfizer, Takeda, Wedbush Securities; received speaker fees from AbbVie, AlfaSigma, Bristol Myers Squibb, CED Service GmbH, Dr. Falk Pharma, Eli Lilly, Galapagos, MD Education, MSD, Galapagos, Janssen/ Johnson&Johnson, Pfizer, Tr1X; and grant support from Pfizer. Carl Weidinger served as a consultant for Pfizer, received speaker's fees from Falk, Ferring, and Janssen, and received grant support from Pfizer. These activities are unrelated to the present study. Toka Omar declares no conflict of interest.

## Data Availability

No datasets were generated or analyzed for this study. All data supporting the conclusions of this review are available in the cited literature.
